# Arsenic and Prostate Cancer: Acquiring Androgen Independence

**Published:** 2005-09

**Authors:** John Tibbetts

Scientists already suspect that prostate cancer, the second-leading cause of cancer death in U.S. men, is linked with chronic arsenic exposure. Now a team of researchers reports that human prostate cells that underwent chronic, low-level arsenic exposure not only exhibited aggressive carcinoma-like growth, but also showed an increased incidence of androgen independence, a state often linked to advanced or fatal prostate cancers, and one that makes these cancers more difficult to treat **[*EHP *113:1134–1139]**.

Androgen, a sex hormone that stimulates and maintains masculine characteristics, is a necessary component in normal prostate function that can also encourage the survival and growth of prostate cancer. That is why some current treatments, such as pharmaceutical androgen blockers or removal of the testes, focus on making androgen less available to prostate cancer cells. However, some patients experience androgen independence, in which prostate cancer cells no longer need the male hormone to differentiate and grow out of control.

The mechanisms behind androgen independence are not completely understood, but scientists do know that this phenomenon sometimes occurs when androgen receptors go functionally awry. In some cases, the androgen receptors are “overexpressed,” greatly increasing in number. In other cases, the receptors mutate, which can result in hyperresponsiveness to androgens. Still other receptor mutations allow nonandrogens (such as the estrogens) and even androgen inhibitors to stimulate cancer growth. Sometimes androgen receptors are bypassed completely, and cell growth is activated by other naturally occurring compounds including insulin-like growth factor-1, epidermal growth factor, and others.

In the current study, researchers examined the growth of normal and arsenic-transformed human prostate epithelial cells. The arsenic-transformed cells had been exposed continuously to sodium arsenite and, after 29 weeks of exposure, produced malignant tumors when inoculated into nude mice. Both cell lines were observed in two different media. One medium included the complete range of steroids, including ample amounts of androgen and growth factors. The other lacked normal amounts of sex hormones and growth factors.

The experiment showed that, consistent with malignant cell growth, prostate cells chronically exposed to arsenic grew more rapidly than control cells in both media. In the steroid-rich medium, the arsenic-transformed cells proliferated approximately twice as fast as the unexposed control cells. In the steroid-depleted medium, the arsenic-transformed cells proliferated about 2.5 times faster than control cells.

Arsenic exposure therefore appeared to be associated with the acquisition of androgen independence in prostate cells. However, the observed arsenic-induced androgen independence did not occur by any previously studied mechanism; it was not linked to overexpression of androgen receptors or receptor mutations that facilitate cell growth via nonandrogens.

A clue may lie in earlier experiments, in which the same researchers observed a marked increase in production of K-*ras* (an oncogene associated with prostate cancers) in arsenic-transformed cells. K-*ras* is a key part of a growth-stimulating pathway in the prostate that is stimulated by androgens. K-*ras* was clearly correlated with arsenic-induced carcinoma-like growth and androgen independence. The authors speculate here that arsenic may bypass the androgen receptor and directly cause aberrant K-*ras* activation.

